# Supplementary material for: Transcriptomic Analysis Reveals That Rho GTPases Regulate Trap Development and Lifestyle Transition of the Nematode-Trapping Fungus Arthrobotrys oligospora
Source: Microbiol Spectr. 2022 Jan 12;10(1):e01759-21. doi: 10.1128/spectrum.01759-21 (PMC8754127; doi:10.1128/spectrum.01759-21)

SUPPLEMENTAL FIGURES (Fig. S1-S8)

Fig. S1 Spearman correlation coefficient and principal component analyses (PCA) for each sample. (A) The Spearman correlation coefficient estimated for three biological samples at each time point. Spearman correlation coefficient is above 0.92 for WT. (B) PCA analyses of the WT strains were performed on three biological samples at each time point.

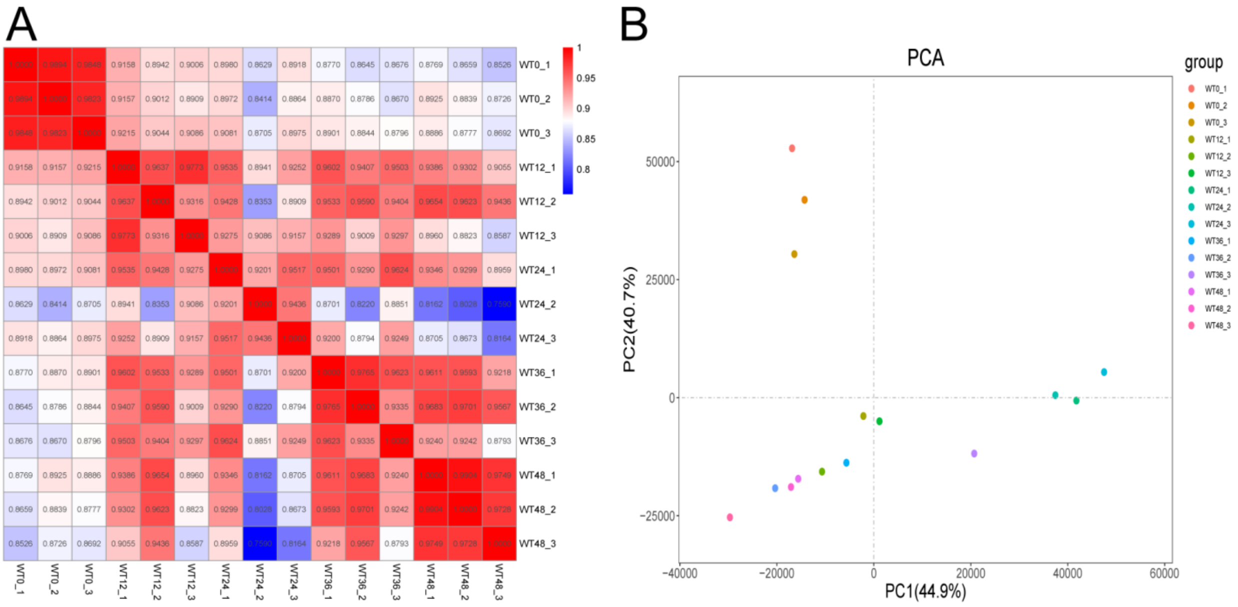

Fig. S2 Verification of the AS events and transcriptional levels of Rho GTPases. (A) Transcriptome alignment identified 3058 to 4488 splicing events in 15 sample libraries, respectively. (B) Verification of the AS events in *Aorho2* using RT-PCR; a. The diagrammatic representation of the *Aorho2* gene. b. The AS events of gene *Aorho2* were verified using RT-PCR. The target cDNA was amplified using AoRho2-P1 and AoRho2-P2 primers, and the AS events were verified using AoRho2-P2/AoRho2-P3 and AoRho2-P4/AoRho2-P5 primers (Table S9). M1K represents the 1000 DNA marker, M500 represents the 500 DNA marker. (C) Verification of the AS events in *Aorac* using RT-PCR; a. The diagrammatic representation of the *Aorac* gene. b. The AS events of gene *Aorac* were verified using RT-PCR. AoRac-P1 and AoRac-P2 primers were used to amplify the target cDNA, and AoRac-P1and AoRac-P3 primers (Table S9) were used to verify the AS events. M1K represents the 1000 DNA marker, M500 represents the 500 DNA marker. (D) Relative transcript levels (RTLs) of *Aorho2*, *Aocdc42*, and *Aorac* genes in the WT strain were monitored during the trap formation and nematode predation on water agar medium at different time points. The red line indicates the standard (with RTL=1) for statistical analysis of the RTL of each gene in the WT strain under a given condition. Error bars in (D) show SD. Asterisk indicates a significant difference (n = 3 for each gene; Tukey's HSD, *P*<0.05).

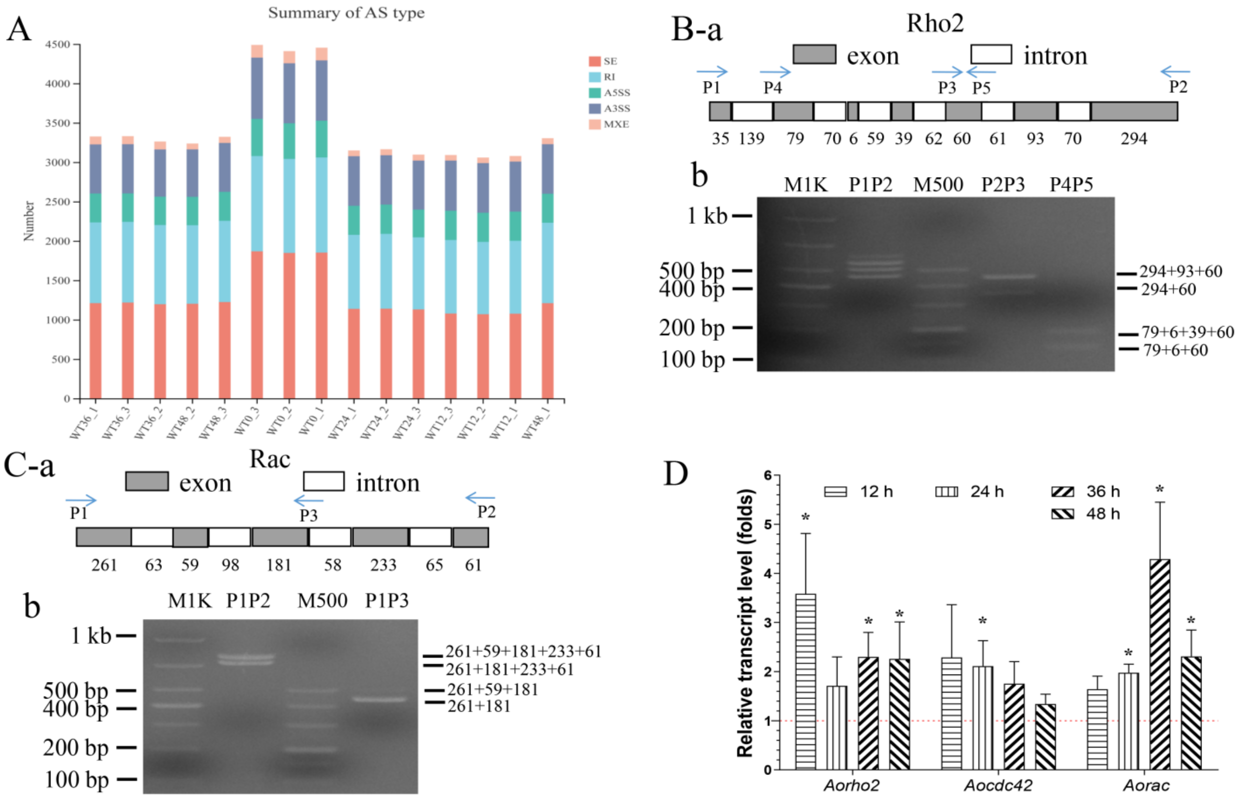

Fig. S3 Multiple sequence alignment of Rho GTPases. The amino acid sequences of Rho GTPases from different fungi are aligned by DNAman software. Areas shaded in black represent conserved regions (100% similarity), areas shaded in red represent high degree similarity (more than 75% similarity), and those shaded in yellow show middle degree similarity (more than 50% similarity), while unshaded areas are regions of variability between the Rho2, Cdc42, and Rac.

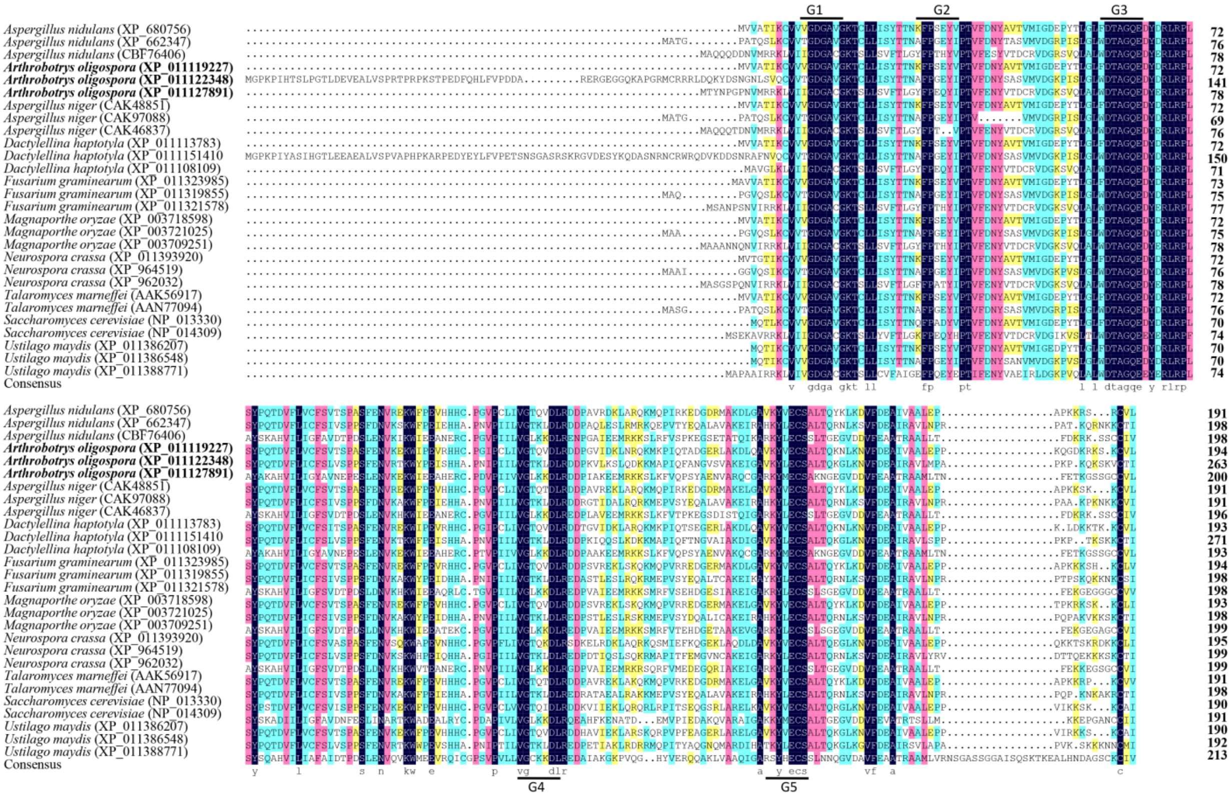

Fig. S4 Three-dimensional structure models of Rho GTPases and phylogenetic analyses of Rho GTPases. (A)The three-dimensional structures of Rho GTPases in *A. oligospora*are predicted by Iterative Threading Assembly Refinement (I-TASSER). (B) Phylogenetic relationship among orthologous Rho2, Cdc42, and Rac proteins from *A. oligospora* and other fungi. The orthologs of Rho2 (AoRho2), Cdc42 (AoCdc42), and Rac (AoRac) in *A. oligospora* are marked in blue color.

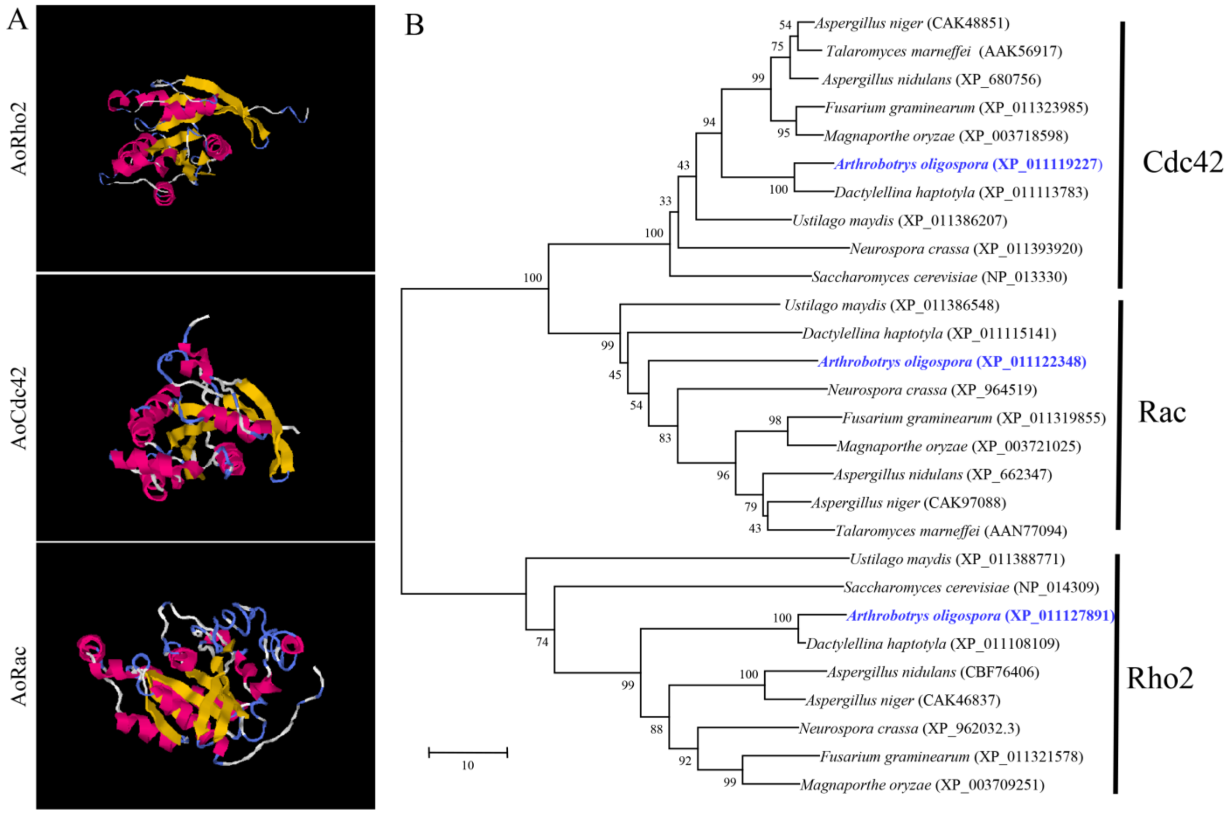

Fig. S5 Verification of the knockdown of Rho GTPase genes in *A. oligospora*. (A) Verification of the *Aorho2* gene knockout using PCR and Southern blot analysis. a. The diagrammatic representation of homologous recombination of the *Aorho2* gene. Primers AoRho2-5f/AoRho2-5r and AoRho2-3f/AoRho2-3r were used for the amplification of homologous flanks of the target gene, and the primers AoRho2-Yf/AoRho2-Yr (Table S9) were used for the verification of transformants. Probe (P) indicates the site of the Southern blot probe, and *Xho*I was the restriction enzyme used for Southern blot analysis. b. The transformants of gene *Aorho2* were verified using PCR with the primers AoRho2-Yf/AoRho2-Yr. Line 3, 4, and 5 suggest positive transformants, line 1 suggests the wild-type (WT) strain, whereas line 2 suggests heterozygotic transformant with a WT gene copy and *hph*-replaced copy. M represents the DNA marker. c. Southern blot analysis of the WT and Δ*Aorho2* mutants. MT represents three independent mutants. (B) Verification of the *Aocdc42* gene knockout using PCR and Southern blot analysis. a. The diagrammatic representation of homologous recombination of the *Aocdc42* gene. Primers AoCdc42-5f/AoCdc42-5r and AoCdc42-3f/AoCdc42-3r were used for the amplification of homologous flanks of the target gene, and the primers AoCdc42-Yf/AoCdc42-Yr (Table S9) were used for the verification of transformants. Pindicates the site of the Southern blot probe, and *Bst*EII was the restriction enzyme used for Southern blot analysis. b. The transformants of gene *Aocdc42* were verified using PCR with the primers AoCdc42-Yf/AoCdc42-Yr. Line 3, 4, and 5 suggest positive transformants, line 1 suggests the wild-type (WT) strain, whereas line 2 suggests heterozygotic transformant with a WT gene copy and *hph*-replaced copy. M represents the DNA marker. c. Southern blot analysis of the WT and Δ*Aocdc42* mutants. MT represents three independent mutants. (C) Verification of the *Aorac* gene knockout using PCR and Southern blot analysis. a. The diagrammatic representation of homologous recombination of the *Aorac* gene. Primers AoRac-5f/AoRac-5r and AoRac-3f/ AoRac-3r were used for the amplification of homologous flanks of the target gene, and the primers AoRac-Yf/AoRac-Yr (Table S9) were used for the verification of transformants. P indicates the site of the Southern blot probe, and *Hin*dIII was the restriction enzyme used for Southern blot analysis. b. The transformants of gene *AorheB* were verified using PCR with the primers AoRac-Yf/AoRac-Yr. Line 3, 4, and 5 suggest positive transformants, line 1 suggests the wild-type (WT) strain, whereas line 2 suggests heterozygotic transformant with a WT gene copy and *hph*-replaced copy. M represents the DNA marker. c. Southern blot analysis of the WT and Δ*Aorac* mutants. MT represents three independent mutants.

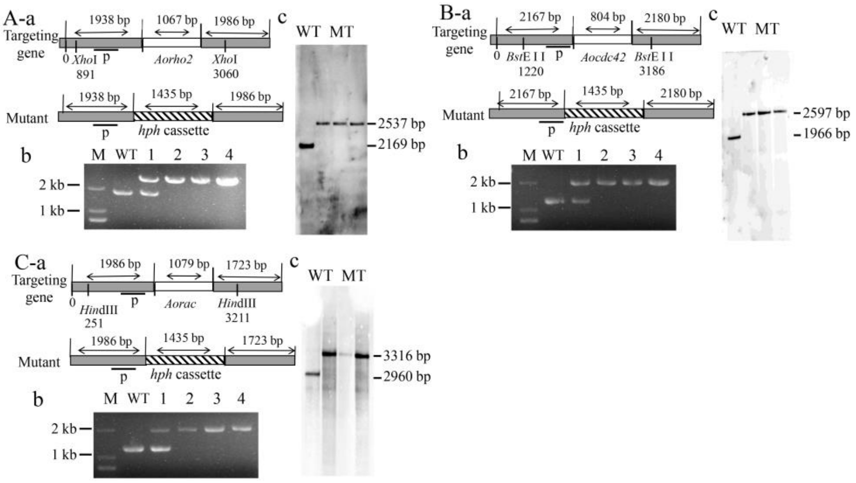

Fig. S6 Comparison of the stress tolerance of strains to oxidative stress. (A) Colony morphologies of the WT and mutant strains under oxidative stress conditions. (B–C) Colony diameters and the relative growth inhibition (RGI) values of the strains cultured in the presence of (B) 5–15 mM H_2O_2 and (C) 0.01–0.05 mM menadione. (D) Relative transcript levels (RTLs) of oxidation-related genes in the mutants when compared with the WT strain at different time points. Red line indicates the standard (which has a RTL of 1) for statistical analysis of the RTL of each gene in the deletion mutant to that in the WT strain under a given condition. Error bars in (B–D): Data are represented as mean ± SD. The asterisk in (B–D) indicates a significant difference between the mutants and the WT strain (n = 3 for the WT strain (B and C), n = 9 for each mutant strain (B and C), n = 3 for each gene (D); Tukey's HSD, P<0.05).

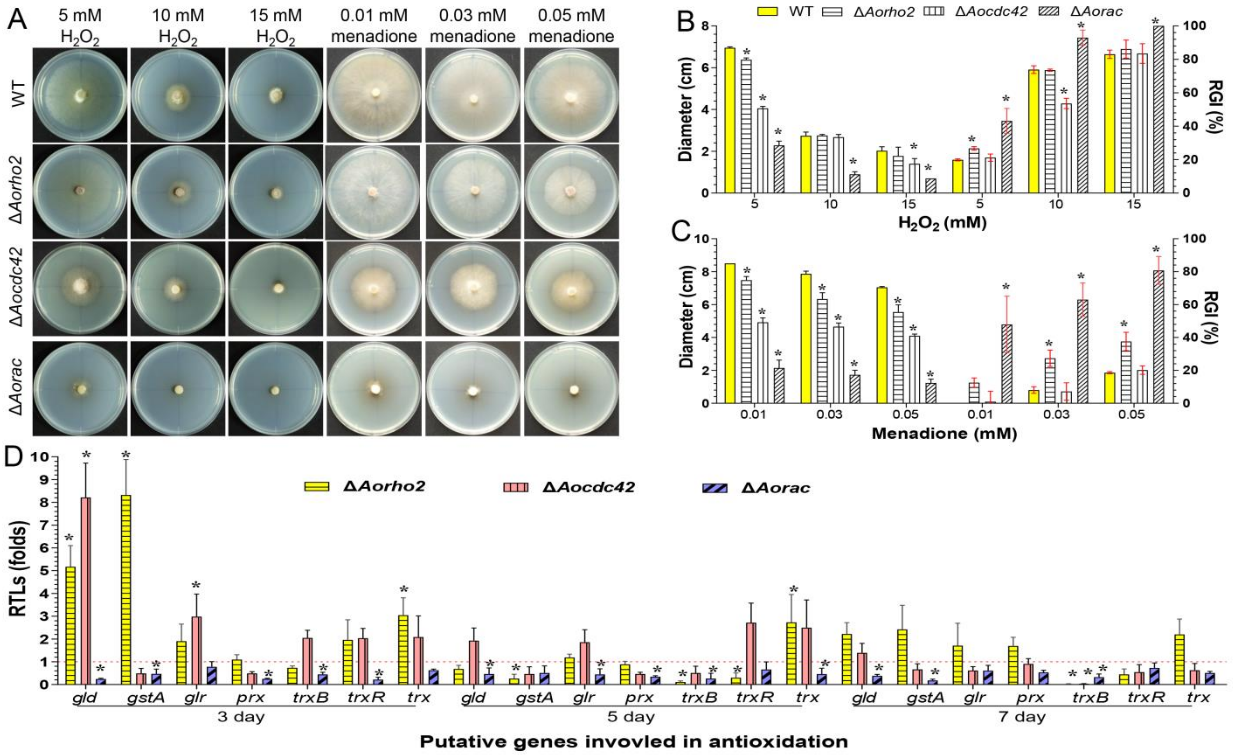

Fig. S7 Comparison of stress tolerance of strains to osmotic agents. (A) Colony morphologies of the WT and mutant strains under stress conditions such as osmotic agents.(B–C) Colony diameters and the relative growth inhibition (RGI) values of the strains cultured in the presence of (B) 0.10–0.30 M NaCl, (C) 0.25–1 M sorbitol. Error bars in (B and C): Data are represented as mean ± SD. The asterisk in (B and C) indicates a significant difference between the mutants and the WT strain (n = 3 for the WT strain (B and C), n = 9 for each mutant strain (B and C); Tukey's HSD, $P<0.05$).

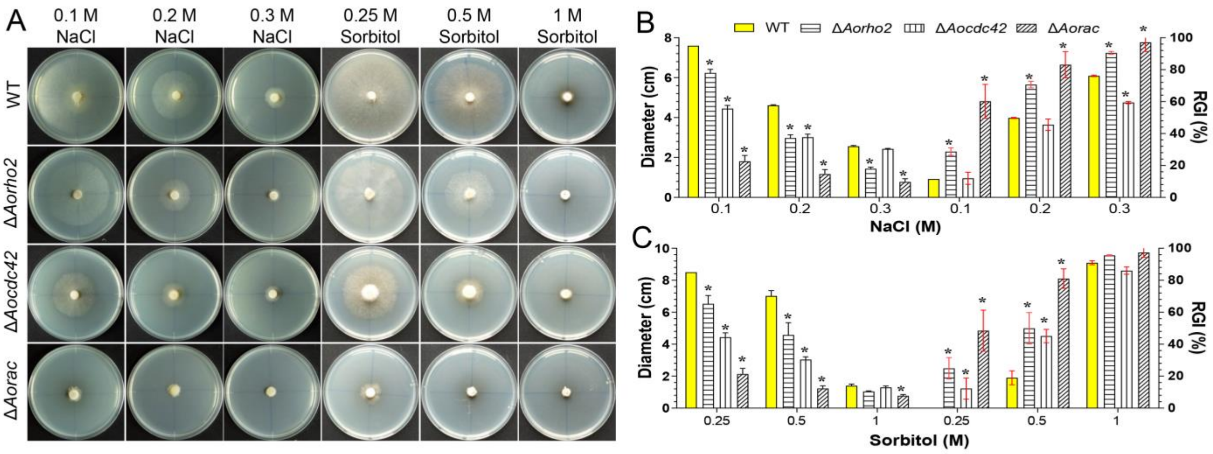

Fig. S8 Comparison of trap formation, nematocidal activity, extracellular proteolytic activity, and ROS production. (A) Trap formation in the WT and Δ*Aorac* mutant strains induced by nematodes at different time points. The red arrows show the traps produced by the WT strain and mutants. Bar = 50 μm. (B) The numbers of traps produced by the WT and Δ*Aorac* mutant strains. Error bars: Data are represented as mean ± SD. The asterisk indicates a significant difference between the mutants and the WT strain (n = 3 for the WT strain, n = 9 for the mutant strain; Tukey's HSD, *P*<0.05).(C) Comparison of the extracellular proteolytic activities on casein plates. (D) Light micrographs of DHE-stained hyphae observed under DIC. The red arrows point to mycelia that do not produce ROS. Bar = 100 μm.

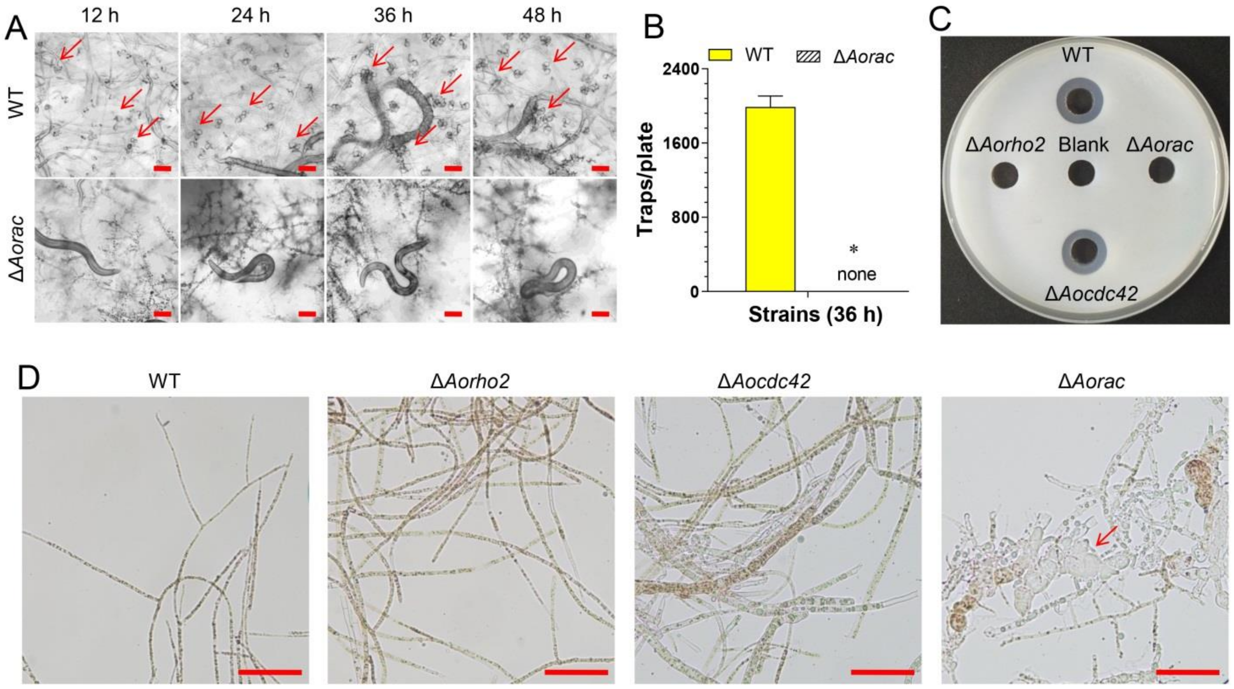

Supplement: SUPPLEMENTAL FILE 1 — Supplemental material. Download SPECTRUM01759-21_Supp_1_seq4.pdf, PDF file, 2.4 MB [file spectrum01759-21_supp_1_seq4.pdf]
